# Prophylactic Inhalation of Bergamot Essential Oil Ameliorates Hormone‐Simulated Pseudopregnancy Withdrawal‐Induced Postpartum Depression by Modulating Hippocampal Synaptic Plasticity

**DOI:** 10.1111/jcmm.71395

**Published:** 2026-10-22

**Authors:** Huan Xu, Shike Zhang, Hui He, Xue Zhao, Yupei Cheng, Yingdong Wang, Jinzhou Li, Yi Yu, Yuhong Bian, Yi Guo, Mingxing Zhang

**Affiliations:** ^1^ School of Integrated Traditional Medicine, Tianjin University of Traditional Chinese Medicine Tianjin P. R. China; ^2^ Research Center of Experimental Acupuncture Tianjin University of Traditional Chinese Medicine Tianjin P. R. China; ^3^ National Clinical Research Center for Chinese Medicine Tianjin P. R. China; ^4^ Department of Reproductive Medicine First Affiliated Hospital of Zhengzhou University Zhengzhou P. R. China

**Keywords:** aromatherapy, bergamot essential oil, hippocampus, neuroplasticity, postpartum depression, prophylactic administration

## Abstract

Postpartum depression (PPD) affects approximately 17.2% of women giving birth globally, but current pharmacotherapies face significant limitations including invasive intravenous delivery (brexanolone) and high costs Bergamot essential oil (BEO) has demonstrated efficacy in aromatherapy for depression, yet its role in PPD remains unexplored. This study investigated whether prophylactic or therapeutic inhalation of nebulized BEO ameliorates PPD‐like phenotypes in a rat model of hormone‐simulated pseudopregnancy (HSP) with abrupt withdrawal, and explored potential mechanisms involving hippocampal synaptic plasticity and neuroinflammation. The main components of BEO were characterized using gas chromatography–mass spectrometry (GC–MS). A rat model of PPD was constructed using the HSP post‐withdrawal method, and animals were randomly divided into control group (Ctrl), sham surgery group (Sham), model group (PPD), fluoxetine group (Flu; 10 mg/kg, i.p., post‐modelling) as a positive control representing first‐line SSRI therapy (brexanolone, though FDA‐approved for PPD, requires costly intravenous administration and specialized medical supervision, and was therefore not used as a comparator), therapeutic BEO group (BEO; 2.5% v/v inhalation post‐modelling) and prophylactic BEO group (P‐BEO; 2.5% v/v inhalation during HSP modelling). The sucrose preference test (SPT), open field test (OFT) and elevated plus maze (EPM) were used to evaluate the depressive‐like behaviours, and HE staining was used to observe the effects of BEO on the thymus and spleen of immune organs. Nissl staining was utilized to quantify the Nissl bodies in the hippocampus (HIPP) of PPD model rats. Additionally, immunohistochemistry and Western blotting were conducted to detect alterations in postsynaptic density protein‐95 (PSD‐95) and synaptophysin (SYP) within the hippocampus of the rats. Enzyme‐linked immunosorbent assay (ELISA) was employed to further analyse the levels of insulin‐like growth factor‐1 (IGF‐1, a neurotrophic factor linked to synaptic plasticity), interleukin‐1β (IL‐1β, a pro‐inflammatory cytokine implicated in PPD pathogenesis) and interleukin‐10 (IL‐10, an anti‐inflammatory cytokine implicated in PPD pathogenesis) in serum, cerebrospinal fluid (CSF) and HIPP. Molecular docking was performed to predict interactions between BEO's core constituents (limonene, γ‐terpinene, α‐pinene, β‐pinene, α‐phellandrene) and target proteins (PSD‐95, SYP, IL‐1β, IGF‐1, IL‐10). Both therapeutic and prophylactic BEO inhalation significantly increased sucrose preference (from 78.78% ± 17.25% to 96.44% ± 2.60% [BEO] and 97.33% ± 0.87% [P‐BEO], *p* < 0.05), total distance travelled in the OFT (from 900.07 ± 523.07 to 1932.64 ± 665.88 cm [BEO] and 1671.51 ± 544.36 cm [P‐BEO]; *p* < 0.05) and open arm time in the EPM (from 16.34 ± 15.44 to 49.91 ± 24.99 s [BEO] and 50.81 ± 16.12 s [P‐BEO]; *p* < 0.05). Both BEO groups exhibited increased Nissl body counts in hippocampal CA1 (from 7.44 ± 1.59 to 18.44 ± 2.60 [BEO] and 19.56 ± 2.07 [P‐BEO]; *p* < 0.05), CA2, CA3 and DG subregions (*p* < 0.05 vs. PPD), as well as increased PSD‐95/SYP protein abundance. Both BEO regimens increased IGF‐1 levels in serum and CSF (*p* < 0.05 vs. PPD), with the P‐BEO group showing a significantly higher serum IGF‐1 levels compared to the Flu group (*p* < 0.05). Additionally, both P‐BEO and BEO groups decreased IL‐1β levels in serum and HIPP, while increasing IL‐10 levels in the CSF (*p* < 0.05 vs. PPD for all). Molecular docking revealed favourable binding affinities (binding energy < −5.0 kcal/mol) between BEO constituents and all five target proteins. No significant histopathological alterations were observed in thymus or spleen following BEO exposure. Collectively, these findings suggest that BEO inhalation may attenuate PPD‐like phenotypes in this rat model, potentially through modulation of hippocampal synaptic plasticity and neuroinflammation via regulation of IGF‐1, IL‐1β and IL‐10. While these results are preliminary, the non‐invasive delivery and observed dual‐phase efficacy suggest that BEO inhalation may represent a more accessible approach worthy of further investigation for PPD management, particularly in resource‐limited settings where current FDA‐approved treatments are unavailable.

## 1 Introduction

Postpartum depression (PPD) represents a substantial global public health issue. Recent findings from The Lancet Global Burden of Disease (GBD) study indicate that depression is the foremost contributor to Years Lived with Disability (YLDs) [1]. Notably, women are at twice the risk of developing depression compared to men [1]. PPD, recognized as the most prevalent psychiatric disorder during the perinatal period, is characterized as a depressive condition manifesting within the first 12 months following childbirth [2, 3] and affects approximately 17.2% of women giving birth worldwide [4]. The clinical presentation of PPD is similar to other types of depression [5]. According to the Mayo Clinic in the United States, the symptoms of PPD are regarded as a subtype of major depressive disorder. The symptoms encompass mood swings, challenges in forming an emotional connection with the infant, social withdrawal, alterations in appetite and sleep patterns, extreme fatigue, reduced interest in activities that were once pleasurable, irritability and thoughts of harming the infant [6]. PPD not only severely impairs maternal parenting and daily life functioning, but can also lead to catastrophic consequences. Studies have shown that approximately 20% of postpartum deaths are associated with PPD‐related suicidal behaviour [7, 8]. In addition, PPD is a significant contributor to disease‐related disability in women [9], with implications for family structure, child psychological development and socioeconomics [10]. Given its high prevalence, multidimensional harms and increasing disease burden, the World Health Organization (WHO) has prioritized PPD prevention and treatment as a priority for maternal and child health, and interdisciplinary intervention strategies are urgently needed to improve maternal and child health.

Emerging research implicates neuroinflammation and disrupted synaptic plasticity as central to PPD pathogenesis [11]. Although the FDA approved the first targeted drug, brexanolone, in 2019 [12], its intravenous delivery method and high cost have limited clinical application [13]. Notably, the mechanism by which the drug exerts its efficacy—in part through inhibiting inflammatory factors including TNF‐α, IL‐6 and IL‐1β [14, 15, 16]—offers valuable insights for the development of innovative therapies targeting PPD [15], highlighting anti‐inflammatory strategies as promising therapeutic avenues. Aromatherapy, mainly bergamot essential oil (BEO), has been employed in the treatment of various diseases in Western countries for several decades [17]. BEO, the active ingredient of BEO, has been confirmed by clinical studies as an effective adjunctive therapy for improving individual mental health and well‐being [18, 19]. Clinical investigations conducted by Chen et al. have demonstrated the efficacy of inhaled BEO aromatherapy in mitigating depressive symptoms among postpartum women [20] and reducing depression, anxiety and stress in geriatric populations [18]. Given the pivotal role of inflammation in PPD and the limitations associated with current anti‐inflammatory medications, our prior research indicates that BEO may exert its antidepressant effects through possible anti‐inflammatory and neuromodulatory pathways [21]. Crucially, BEO inhalation offers advantages of non‐invasive delivery, rapid onset potential (via olfactory pathways) and a favourable safety profile compared to conventional psychotropics.

Given that prenatal depression is an independent risk factor for PPD [22] and that preventive interventions during pregnancy have significant cost‐effective advantages in reducing the incidence of PPD [23], prophylactic BEO inhalation therapy was innovatively chosen as an intervention strategy in this study. The selection of biomarkers in this study was guided by their established roles in PPD pathophysiology and synaptic plasticity. PSD‐95 (postsynaptic density protein‐95) and SYP (synaptophysin) were chosen as markers of synaptic integrity: PSD‐95 is a scaffolding protein critical for glutamatergic synapse maturation, synaptic transmission and plasticity [24, 25], while SYP is a presynaptic vesicle protein essential for neurotransmitter release and synaptic vesicle operation [26]. Hippocampal injury and stress‐induced synaptic dysfunction are characterized by reduced PSD‐95 and SYP expression [27], making them reliable indicators of neuroplasticity changes in depression models. IGF‐1 (insulin‐like growth factor‐1) was selected based on evidence that low maternal serum IGF‐1 during early pregnancy predicts PPD risk in humans [28, 29], and that IGF‐1 administration produces antidepressant‐like effects in animal models [30, 31, 32]. IL‐1β and IL‐10 were selected as representative pro‐inflammatory and anti‐inflammatory cytokines, respectively, given the established role of inflammatory dysregulation in PPD pathogenesis [33, 34, 35], with elevated IL‐1β [16, 36] and reduced IL‐10 [37] consistently associated with PPD severity.

Currently, although BEO has demonstrated clinical potential in mood regulation, there is still a lack of animal experimental evidence for its specific neural mechanisms against PPD. In this study, we used the internationally recognized hormone‐simulated pseudopregnancy (HSP) post‐withdrawal method to establish a rat PPD model, and for the first time established a dual‐phase dosing regimen of prophylactic (prenatal intervention) and therapeutic (postnatal intervention) administration to examine the effects of BEO on depressive behaviours and hippocampal neuroplasticity in PPD rats, with the aim of providing an experimental basis for its clinical application. Our goal is to provide a robust experimental foundation for translating BEO inhalation, particularly its prophylactic application, into a safe and accessible clinical intervention for PPD prevention and management.

## 2 Materials and Methods

### 2.1 Chemicals and Reagents

Tween 80 (T8360) was obtained from Beijing Solarbio Science & Technology Co. Ltd. (Beijing, China). Haematoxylin and eosin staining solution was provided by Beijing Solarbio Biotechnology Co. Ltd. (Beijing, China). Toluidine blue O (T3260) stain was purchased from Sigma‐Aldrich (Shanghai, USA). PSD‐95 polyclonal antibody (ab18258), SYP monoclonal antibody (ab32127) and β‐actin monoclonal antibody (ab115777) were provided by Abcam (Cambridge, UK). Immunohistochemistry (ICH) staining kit (PK10006) was provided by Proteintech Group Inc. (Wuhan, China). Enzyme‐linked immunosorbent assay (ELISA) kits for IGF‐1 (EK0377), IL‐1β (EK0393) and IL‐10 (EK0398) were purchased from Bost Bio‐Technology Co. Ltd. (Wuhan, China).

### 2.2 Extraction and Identification of BEO

Fresh bergamot fruits were provided by Zhejiang Jinhua Rulai Bergamot Co. Ltd. BEO was extracted by steam distillation and separated by centrifugation. Chemical characterization was performed using gas chromatography–mass spectrometry (GC–MS) [38] on a TG‐5MS capillary column (30 m × 0.25 mm × 0.25 μm). The GC oven temperature was programmed: initial 50°C (hold 3 min), ramp at 5°C/min to 280°C (hold 5 min) (total analysis time of 54 min, carrier gas: He). Injection port temperature was 280°C in split mode (100:1). Compound identification combined GC–MS data with Kovats retention indices, matching mass spectra against the National Institute of Standards and Technology (NIST) 2014 and Wiley 2008 libraries.

### 2.3 Animal and Experimental Design

Healthy female Sprague Dawley rats (50 days old, 180–200 g, SPF grade) from Beijing VTLH Experimental Animal Technology Co. Ltd. (Licence No.: SCXK (Jing) 2021‐0011) were housed under standard SPF conditions (temperature 24.5°C ± 0.5°C, humidity 45%–55%, 12 h/12 h light/dark cycle) at Tianjin University of Traditional Chinese Medicine (TCM). They had free access to water and standard laboratory chow, with a 5‐day acclimatization period prior to experiments. The experimental protocol strictly adhered to China's ‘Regulations on the Management of Laboratory Animals’ and the guidelines provided by the U.S. National Institutes of Health (NIH) for the care and use of laboratory animals. All procedures involving the rats were in compliance with relevant ethical standards and received approval from the TCM Animal Ethics Committee (Approval No. TCM‐LAEC2015033). Rats were excluded based on sucrose preference tests (SPTs), and the remaining rats were randomly assigned to the following six groups (*n* = 9 per group). Control group (Ctrl): No surgical intervention, no hormone administration and no treatment; received inhalation of vehicle solution (1% Tween 80 in distilled water) for 1 h daily during the corresponding experimental period. Sham surgery group (Sham): Underwent bilateral dorsal incisions to expose the ovaries without removal, followed by disinfection and suturing; received no hormone administration; received inhalation of vehicle solution (1% Tween 80 in distilled water) for 1 h daily. Model group (PPD): Underwent ovariectomy (OVX) and HSP with abrupt withdrawal; received inhalation of vehicle solution (1% Tween 80 in distilled water) for 1 h daily for 4 weeks after model confirmation. Fluoxetine group (Flu): Underwent OVX and HSP with abrupt withdrawal; received intraperitoneal injection of fluoxetine (10 mg/kg/day, diluted in physiological saline) for 4 weeks after model confirmation; did not receive inhalation treatment. Therapeutic BEO group (BEO): Underwent OVX and HSP with abrupt withdrawal; received inhalation of 2.5% (v/v) BEO emulsion for 1 h daily for 4 weeks after model confirmation. Prophylactic BEOgroup (P‐BEO): Underwent OVX and HSP with abrupt withdrawal; received inhalation of 2.5% (v/v) BEO emulsion for 1 h daily from Day 1 of HSP modelling through Day 23 (covering the entire modelling period). Body weight was monitored weekly throughout the experiment as an indicator of general health. However, no significant differences were observed among groups at any time point (*p* > 0.05, data not shown), and thus these data are not presented in the main figures. The absence of body weight changes suggests that neither the ovariectomy procedure, hormone administration, nor BEO inhalation induced substantial metabolic disturbances or systemic toxicity in the animals.

### 2.4 Establishment of the PPD Model and Intervention Protocol

Referring to previous studies [39, 40, 41], the procedure first established an HSP model, which was subsequently followed by the abrupt cessation of hormone administration to induce PPD. The experimental timeline is illustrated in Figure 1.

**FIGURE 1:**
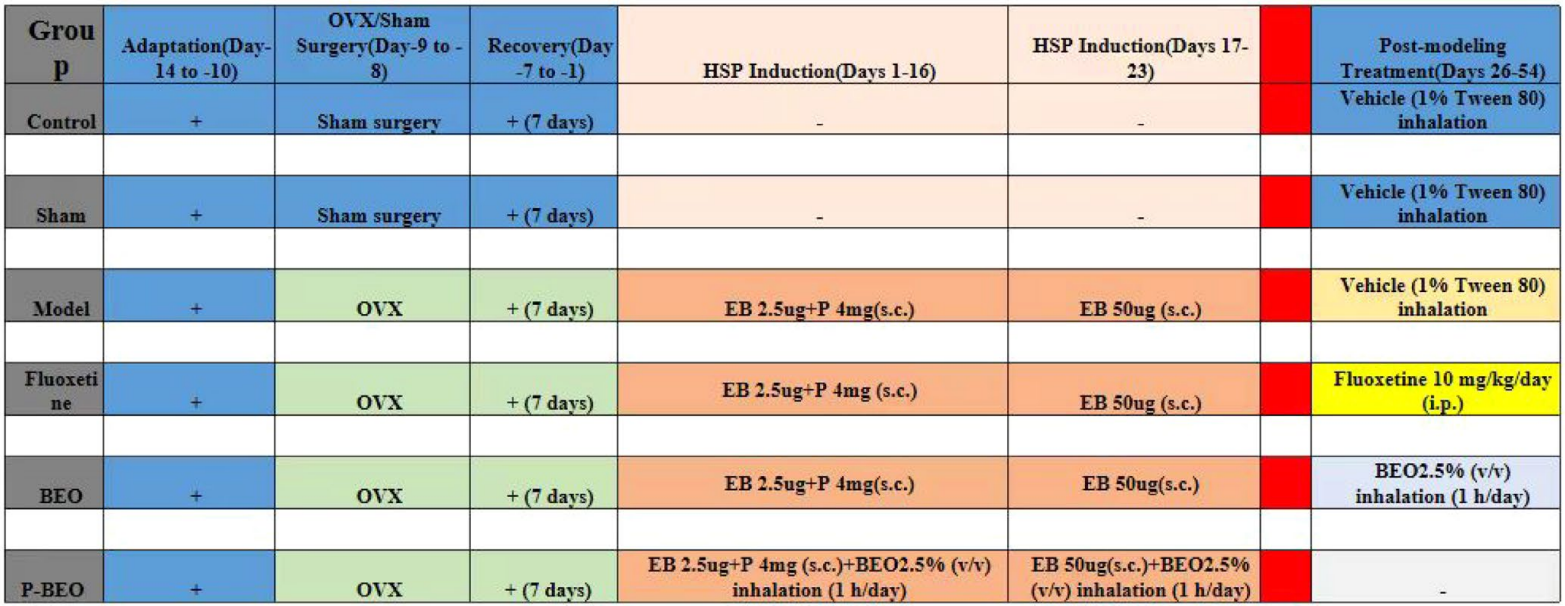
Schematic diagram of the experimental timeline and treatment protocols. After a 5‐day adaptation period (Day −14 to Day −10), rats in the PPD, Flu, BEO and P‐BEO groups underwent bilateral ovariectomy (OVX) on Days −9 to −8, followed by a 7‐day recovery period (Day −7 to Day −1) to eliminate endogenous oestrogen and progesterone. The sham group underwent sham surgery, and the control group received no surgical intervention. Hormone‐simulated pseudopregnancy (HSP) was induced from Days 1 to 23. From Days 1 to 16, OVX rats received daily subcutaneous injections of low‐dose oestradiol benzoate (EB, 2.5 μg/rat/day) and progesterone (P, 4 mg/rat/day). From Days 17 to 23, they received high‐dose EB (50 μg/rat/day). All hormones were dissolved in sesame oil (100 μL per injection). Hormone withdrawal occurred on Days 24–25. To verify successful PPD model establishment, behavioural tests (sucrose preference test, SPT; open field test, OFT; and elevated plus maze, EPM) were performed on Day 26 (2 days after hormone withdrawal). The same behavioural tests were repeated at Weeks 2 and 4 of treatment to evaluate the therapeutic effects of BEO and fluoxetine. The P‐BEO group received BEO inhalation (2.5% v/v, 1 h/day) prophylactically during Days 1–23 (entire HSP period). The BEO and Flu groups began treatment after model validation on Day 26 and continued for 4 weeks. The Flu group received fluoxetine (10 mg/kg/day, i.p.). All animals were sacrificed on Day 67 for tissue collection. BEO, bergamot essential oil; EB, oestradiol benzoate; Flu, fluoxetine; HSP, hormone‐simulated pseudopregnancy; i.p., intraperitoneal; OVX, ovariectomy; P, progesterone; P‐BEO, prophylactic BEO; PPD, postpartum depression; s.c., subcutaneous; v/v, volume/volume.

After a 5‐day adaptation period (Day −14 to Day −10), rats in the PPD, Flu, BEO and P‐BEO groups underwent bilateral ovariectomy (OVX) under isoflurane anaesthesia (3% induction, 1.5% maintenance) on Days −9 to −8. Briefly, ovaries were exposed through bilateral dorsal incisions, the ovarian blood supply was ligated and the ovaries were removed. The control group did not receive any treatment. The sham group underwent bilateral dorsal incisions to locate the ovaries without removal, followed by disinfection and suturing. Following surgery, all rats were allowed a 7‐day recovery period (Day −7 to Day −1) to eliminate endogenous oestrogen and progesterone, during which local penicillin was administered to prevent infection [39].

*Hormone Administration*: From Days 1 to 16, OVX rats received daily subcutaneous injections of low‐dose oestradiol benzoate (2.5 μg/rat/day, approximately 12.5 μg/kg/day based on mean body weight of 200 g) and high‐dose progesterone (4 mg/rat/day, approximately 20 mg/kg/day), both dissolved in sesame oil (100 μL per injection). From Days 16 to 23, they received high‐dose oestradiol benzoate (50 μg/rat/day, approximately 250 μg/kg/day) daily to simulate the hormonal state of pregnancy in rats. Following the final injection on Day 23, hormone administration was abruptly discontinued, initiating a 2‐day hormone withdrawal period (Days 24–25). Behavioural assessments commenced 2 days post‐withdrawal (Day 26) to verify successful PPD model establishment.

*Inhalation Treatment*: BEO emulsion (2.5% v/v) was prepared fresh daily by mixing 3 mL BEO, 120 mL distilled water and 1.2 mL Tween 80. The vehicle solution (1% Tween 80) was prepared by mixing 1.2 mL Tween 80 with 120 mL distilled water. All inhalation treatments were administered for 1 h daily using a homemade nebulizer chamber (20 × 20 × 30 cm) with continuous airflow.

*Inhalation Treatment*: BEO emulsion (2.5% v/v) was prepared fresh daily by mixing 3 mL BEO, 120 mL distilled water and 1.2 mL Tween 80. The vehicle solution (1% Tween 80) was prepared by mixing 1.2 mL Tween 80 with 120 mL distilled water. All inhalation treatments were administered for 1 h daily using a homemade nebulizer chamber (69 × 69 × 24 cm) with continuous airflow.

*Treatment Protocols*: Control Group (Ctrl): No surgical intervention, no hormone administration; received inhalation of vehicle solution (1% Tween 80) for 1 h daily during the corresponding experimental period. Sham surgery group (Sham): Underwent sham surgery; received no hormone administration; received inhalation of vehicle solution (1% Tween 80) for 1 h daily. Model group (PPD): Underwent OVX and HSP with abrupt withdrawal; received inhalation of vehicle solution (1% Tween 80) for 1 h daily for 4 weeks after model confirmation. Fluoxetine group (Flu): Underwent OVX and HSP with abrupt withdrawal; received daily intraperitoneal injections of fluoxetine (10 mg/kg/day, diluted in physiological saline) for four consecutive weeks beginning after successful model confirmation (i.e., after behavioural testing on Day 26); did not receive inhalation treatment. Therapeutic BEO group (BEO): Underwent OVX and HSP with abrupt withdrawal; received inhalation of BEO emulsion (2.5% v/v) for 1 h daily for 4 weeks after model confirmation (i.e., after behavioural testing on Day 26). Prophylactic BEO group (P‐BEO): Underwent OVX and HSP with abrupt withdrawal; received inhalation of BEO emulsion (2.5% v/v) for 1 h daily from Day 1 of the HSP modelling period through Day 23 (covering the entire modelling duration).

*Behavioural Testing Schedule*: For the BEO and Flu groups, behavioural testing (SPT, open field test [OFT], elevated plus maze [EPM]) was conducted at Weeks 2 and 4 of treatment. For the P‐BEO group, behavioural testing was conducted at the same post‐modelling time point as the initial confirmation test (i.e., on Day 26, 2 days after hormone withdrawal).

### 2.5 Sucrose Preference Test

The SPT is designed to evaluate anhedonia by assessing the preference of rats for sweet solutions, thereby reflecting their inclination towards pleasurable experiences [42]. All rats were housed individually, and the test was divided into three stages: ① Adaptation Phase: Rats were provided with two identical bottles containing a 1% sucrose solution for a duration of 24 h. ② Deprivation Phase: Following the adaptation phase, rats underwent a 24‐h period of food and water deprivation. ③ Testing Phase: Rats were given access to normal food and two pre‐weighed bottles—one containing a 1% sucrose solution and the other containing pure water. After 12 h of unrestricted access, the bottles were re‐weighed. The SPT is positively correlated with hedonic state; lower values indicate more severe anhedonia. Sucrose preference was calculated as:

$$\mathrm{SPT}\;\left(\%\right)=\frac{\text{Sucrose solution consumption}\;\left(g\right)}{\text{Sucrose solution consumption}\;\left(g\right)+\text{Pure water consumption}\;\left(g\right)}\times 100\%$$

### 2.6 Open Field Test

A slightly modified OFT was employed to assess the effects of BEO on depression model rats [43]. The OFT was conducted in a sound‐attenuated room. Each rat was placed at a fixed starting position in a black open‐field arena (40 × 40 × 40 cm). Video recording and timing were initiated immediately. Rat activity was recorded for 6 min (2 min habituation + 4 min test). After each rat was tested, the arena was sanitized with 75% ethanol to eliminate odours and other variables that could influence the outcomes of subsequent tests. Parameters analysed included: Total distance travelled (m); Distance in periphery (m); Immobility time (s); Movement time (s). Finally, data analysis was analysed using Etho Vision XT13.0 (Noldus Information Technology).

### 2.7 Elevated Plus Maze

The EPM test, a validated method for assessing anxiety‐related behaviour in rodents, was performed according to established protocols [44]. The EPM exploits rats' curiosity to explore new environments (open arms) and their natural preference for darkness (enclosed arms), with the conflicting behaviours of exploration and avoidance occurring between the two, thereby inducing anxiety. The rats were positioned on the central platform facing a closed arm, and their behaviour was observed for a total of 6 min (1 min for habituation and 5 min for testing). The maze underwent cleaning with 75% ethanol after every trial. Increased open arm exploration correlates negatively with anxiety levels. Finally, the data were analysed using Etho Vision XT 13.0 (Noldus Information Technology).

### 2.8 Histological Staining (H&E)

Paraffin sections of rat thymus and spleen were processed as follows: Dewaxing: Immersed in Xylene I (5 min) → Xylene II (5 min); Hydration: Gradient ethanol series: 100% → 95% → 75% (5 min each); Rinsing: Washed in running tap water (5 min), then soaked (5 min); Haematoxylin staining: Immersed in Mayer's haematoxylin (5–10 min); Washing: Rinsed under running water (10 min); Differentiation: Treated with 1% acid alcohol (3 s), rinsed until nuclei turned blue; Eosin staining: Counterstained in eosin Y (3–5 s), washed to remove excess dye; Dehydration: Reverse ethanol gradient (75% → 95% → 100%, 2 min each); Clearing: Xylene (2 changes × 3 min); Mounting: Coverslipped with neutral balsam.

### 2.9 Molecular Docking

2D structures of BEO components were obtained from PubChem and energy‐minimized (ChemBio3D). Protein structures (PSD‐95, SYP, IL‐1β, IGF‐1, IL‐10) were retrieved from the RCSB PDB database, prepared (water/ligand removal, hydrogen addition) using PyMol and AutoDockTools and converted to PDBQT format. Docking was performed using AutoDock, and results were visualized with PyMol.

### 2.10 Nissl Staining

Nissl staining was performed to assess morphological changes in hippocampal neuronal Nissl bodies. Paraffin‐embedded brain tissue fixed with 4% paraformaldehyde for more than 48 h (with the fixative replaced every 24 h) was sectioned into 5 μm thick consecutive slices and mounted on slides. Prior to staining, the sections were subjected to dewaxing and graded ethanol hydration: the sections were placed in Xylene I and II for 5 min each to remove paraffin, followed by a graded ethanol series of 100% → 95% → 75% ethanol and finally in double‐distilled water. Immerse the hydrated sections in a toluidine blue staining solution and incubate at 56°C in a constant‐temperature oven for 1 h. After staining, thoroughly remove any residual dye solution and rinse three times with double‐distilled water (3 min each). Subsequently, perform ethanol dehydration using a gradient: immerse the sections in 75% ethanol (5 min) → 95% ethanol (5 min) → 100% ethanol (under microscopic monitoring until background became optically clear). Monitor dynamically under a microscope until the background tissue is completely decoloured (appearing optically clear), then immediately terminate the process. After dehydration, the sections were treated with Xylene I and II for 5 min each, followed by neutral resin mounting. When covering the coverslip, strict care was taken to avoid the formation of bubbles. The staining results were observed under an optical microscope (Nikon, Y‐TV55) to examine the morphological characteristics and quantitative changes of Nissl bodies in the hippocampus.

### 2.11 Immunohistochemistry

ICH was conducted to assess the expression of SYP and PSD‐95, key proteins in hippocampal synaptic plasticity. Paraffin sections were sequentially dewaxed with Xylene I and II for 20 min each, followed by a dehydration process employing a graded ethanol series (100%, 95%, 80% and 60%), with each concentration applied for 5 min, and subsequently rinsed with running water for 3 min. The sections were subjected to microwave‐induced antigen retrieval using sodium citrate buffer (medium heat boiling, 95°C, repeated three times, each for 5 min), then cooled naturally and rinsed three times with PBS buffer (each for 1 min). Endogenous peroxidase was blocked by 3% H_2_O_2_ incubation and rinsed with PBS. Blocking: Incubated with 5% blocking serum at room temperature for 1 h. Primary antibody: Incubated with anti‐SYP/PSD‐95 antibodies (4°C, overnight) → washed with PBS. HRP‐labelled polymer was applied for secondary antibody and incubated at room temperature for 30 min. After washing with PBS, 15 μL DAB was added → colour reaction was monitored microscopically (5–10 min) → stopped by distilled water rinsing. Counterstaining: Stained with haematoxylin for 2 min, rinsed with running water for 3 min. Dehydration: Processed through graded ethanol and cleared with xylene twice (5 min each). Mounting: Coverslipped with neutral balsam. Observed the localization and intensity of specific protein expression in the hippocampus under an optical microscope. The quantification of positive staining area of PSD‐95 and SYP was quantified using ImageJ 1.45 software to quantitatively measure. Quantification: All immunohistochemical analyses were performed by investigators blinded to group allocation. For quantification, five coronal sections per animal (spaced 200 μm apart) spanning the hippocampal formation (bregma −2.0 to −4.5 mm) were selected. The regions of interest were defined as CA1, CA2, CA3 and DG subregions. Images were captured under identical light intensity and exposure settings using a Nikon Y‐TV55 microscope with a 200× objective. The integrated optical density (IOD) of PSD‐95 and SYP immunoreactivity was measured using ImageJ 1.45 software after background subtraction with a standardized threshold. IOD values were averaged across the five sections per animal and expressed as mean IOD per ROI.

### 2.12 Western Blot Detection of Protein Blotting

Protein concentrations were quantified with the BCA assay following the homogenization of HIPP tissues in RIPA buffer. After being resolved by SDS‐PAGE, proteins were transferred onto PVDF membranes and subjected to blocking. The membranes were incubated overnight with primary antibodies specific to PSD‐95 (dilution 1:2000) and SYP (dilution 1:10,000). Following washes with TBST, detection involved incubating the membranes with a secondary antibody linked to HRP, diluted at 1:10,000. Quantification: Western blot band intensities were quantified using ImageJ 1.45 software. All samples were coded and analysed in a blinded manner; the code was broken only after data analysis was complete. PSD‐95 and SYP protein expression levels were normalized to β‐actin (loading control) and expressed as fold‐change relative to the Ctrl group. Each sample was run in duplicate on separate gels to ensure reproducibility.

### 2.13 Enzyme‐Linked Immunosorbent Assay

The levels of IGF‐1, IL‐1β and IL‐10 in serum, cerebrospinal fluid (CSF) and HIPP of HSP‐induced rats were measured using validated ELISA kits, following the instructions provided by the manufacturer. Optical density at 450 nm was measured using a microplate reader (Thermo Fisher, USA).

### 2.14 Statistical Analysis

Data are shown as mean ± SEM. SPSS 26.0 was used for statistical analysis. Normality and variance homogeneity were checked initially using the Shapiro–Wilk test and Levene's test, respectively. Data meeting these criteria were analysed with one‐way ANOVA followed by Tukey's post hoc test for group comparisons. For data not meeting these criteria, the Kruskal–Wallis test followed by Dunn's post hoc test was applied. Statistical significance was set at *p* < 0.05 (two‐tailed). All quantitative analyses (IHC, Western blotting, ELISA) were performed by investigators blinded to group allocation as described in Sections 2.11 and 2.12.

## 3 Results

### 3.1 GC–MS Analysis

BEO from the same batch as our previous study [21], was analysed by GC–MS to characterize its chemical composition. The chromatographic profile is presented in Figure 2 (adapted from [21]). GC–MS analysis identified 15 constituents, with key components comprising: limonene (61.69%), γ‐terpinene (28.02%), α‐pinene (3.52%), β‐pinene (2.80%) and α‐phellandrene (1.28%) [21]. Minor constituents included: β‐myrcene (0.30%), camphene (0.32%), *p*‐cymene (0.28%), α‐terpinene (0.44%), caryophyllene (0.23%) and others (≤ 0.25% each) [21]. The chemical structures of the five major constituents are shown in Figure 2. Collectively, these five major constituents accounted for 97.31% of total volatile content, consistent with previous reports [45].

**FIGURE 2:**
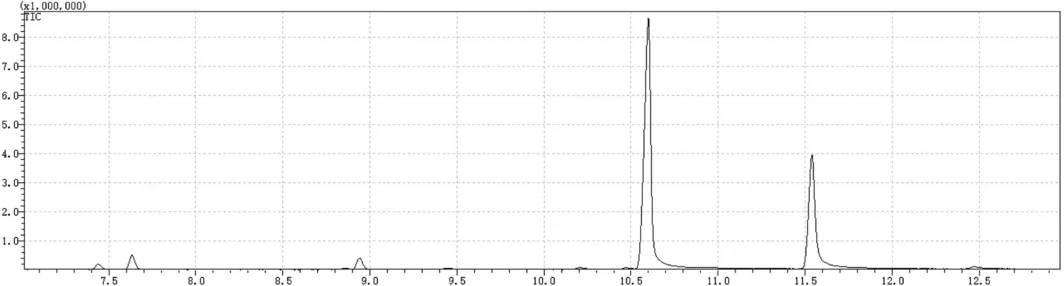
GC–MS total ion chromatogram of BEO. The chromatogram was obtained from the same BEO batch as described in our previous study [21]. Major peaks are labelled: (1) α‐pinene; (2) β‐pinene; (3) limonene; (4) γ‐terpinene; and (5), α‐phellandrene. The five major constituents accounted for 97.31% of the total volatile content.

### 3.2 Validation of Hormone Withdrawal‐Induced PPD Model

Behavioural assessments (SPT, OFT and EPM) were performed 48 h post‐hormone withdrawal. No significant differences were observed between the Control and Sham groups in any of the behavioural tests (*p* > 0.05 for all parameters), confirming that the surgical procedure itself did not induce behavioural alterations. Compared to both the controls and Sham groups, PPD model rats exhibited significantly decreased sucrose preference (*p* < 0.05), reduced total locomotion distance in the OFT (*p* < 0.05), decreased open arm time in the EPM (*p* < 0.05) and increased closed arm time (*p* < 0.05). In contrast, the Flu and BEO groups showed behavioural parameters comparable to the Control and Sham groups (*p* > 0.05 for all comparisons). The P‐BEO group also showed significant improvements compared to the PPD group, with increased sucrose preference, increased total locomotion, increased open arm time and decreased closed arm time (all *p* < 0.05 vs. PPD). No significant differences were observed among the Flu, BEO and P‐BEO groups (*p* > 0.05 for all intergroup comparisons). These results confirm successful PPD modelling and indicate that both therapeutic and prophylactic BEO administration exert antidepressant‐like effects (Figure 3).

**FIGURE 3:**
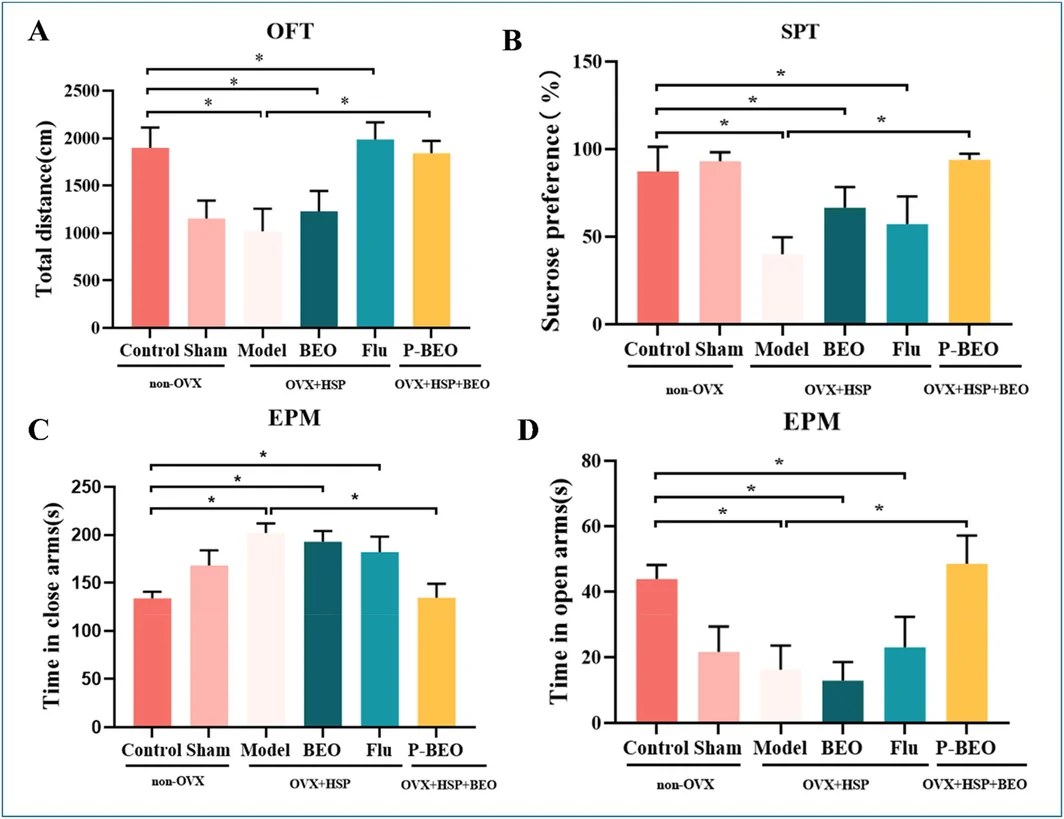
Constructing a PPD model for hormone withdrawal. (A) The impact of hormone withdrawal on open field experiments. (B) The effect of hormone withdrawal on sugar water preference experiment. (C) The effect of hormone withdrawal on the elevated cross maze experiment (open arm dwell time). (D) The effect of hormone withdrawal on the elevated cross maze experiment (closed arm dwell time) (*p < 0.05).

### 3.3 BEO Ameliorates Depressive‐Like Behaviours in PPD Model Rats

The SPT sensitively measures anhedonia, a core depressive symptom. One‐way ANOVA revealed significant group differences in sucrose preference (*p* < 0.001). Post hoc analysis showed that the PPD group had significantly lower sucrose preference than the Control group (*p* < 0.05) and the Sham group (*p* < 0.05). The BEO, P‐BEO and Flu groups all exhibited significantly higher sucrose preference than the PPD group (*p* < 0.05 for all). No significant differences were observed among the BEO, P‐BEO and Flu groups (*p* > 0.05 for all pairwise comparisons), nor between these three groups and the Control or Sham groups (*p* > 0.05) (Figure 4A).

**FIGURE 4:**
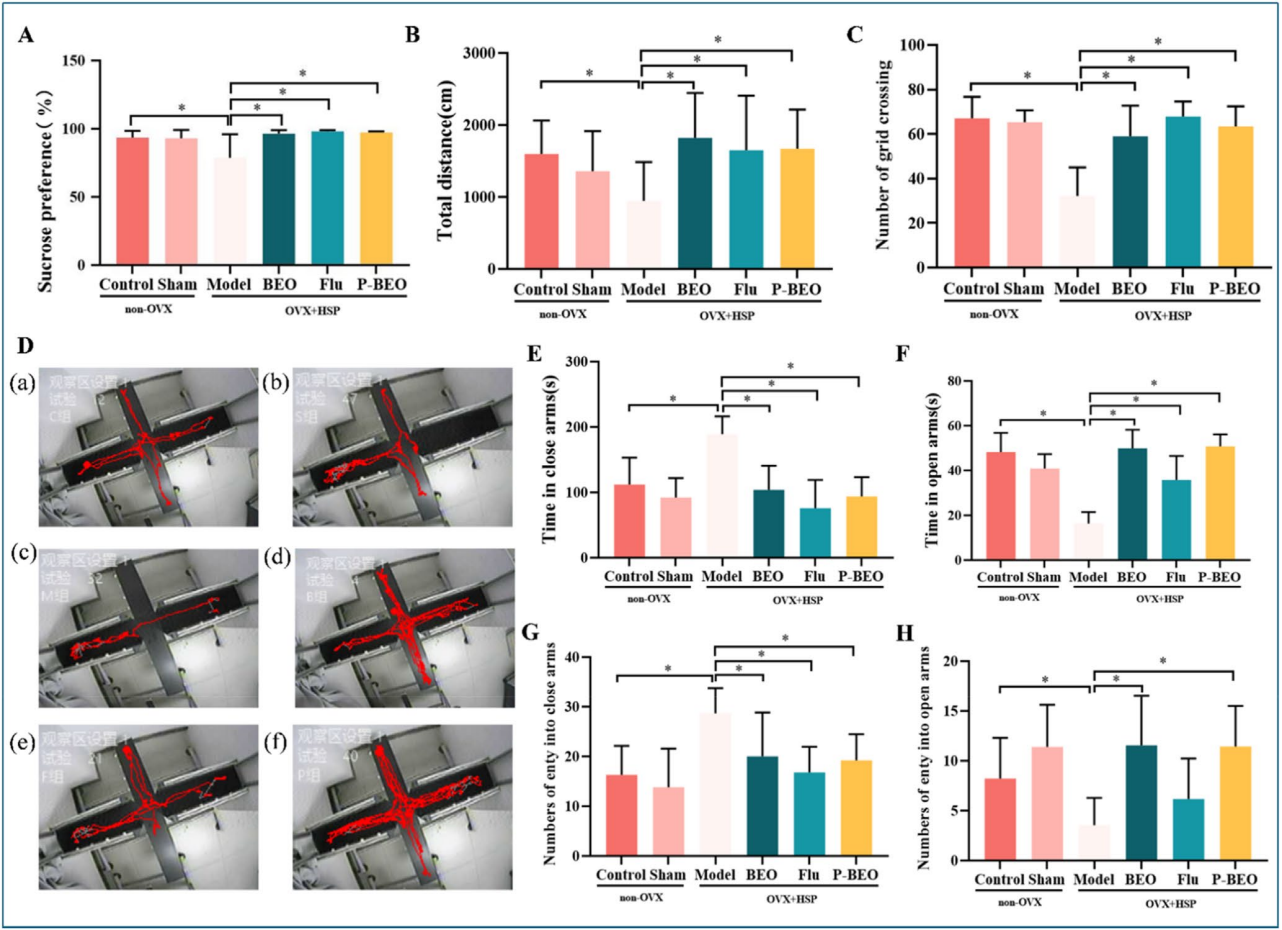
BEO improves depression like behaviour in PPD model rats. (A) The effect of BEO on sucrose preference experiment in PPD model rats. (B) The effect of BEO on the open field experiment of PPD model rats (total distance travelled). (C) The effect of BEO on the open field experiment of PPD model rats (number of spans). (D) Schematic diagram of typical movement trajectories of rats in each group of elevated cross maze (a: Control group; b: Sham group; c: PPD group; d: BEO group; e: Flu group; f: P‐BEO group). (E) Closing arm dwell time. (F) Open arm dwell time. (G) Number of times entering the closed arm. (H) Number of times to enter the open arm (*p < 0.05).

OFT assessments revealed: Significant group differences were observed in total distance travelled (*p* < 0.001) and grid crossings (*p* < 0.001). The PPD group showed significantly reduced total distance and grid crossings compared to both the Control and Sham groups (*p* < 0.05). The BEO, P‐BEO and Flu groups showed significantly increased total distance and grid crossings compared to the PPD group (*p* < 0.05). No significant differences were detected among the BEO, P‐BEO and Flu groups (*p* > 0.05) (Figure 4B,C).

EPM analyses showed: PPD group: The PPD group exhibited significantly decreased open arm entries and open arm time, and significantly increased closed arm entries and closed arm time compared to the Control and Sham groups (all *p* < 0.05). The BEO and P‐BEO groups reversed all these deficits (*p* < 0.05 vs. PPD). The Flu group showed significantly increased open arm time and decreased closed arm entries and time compared to the PPD group (*p* < 0.05). No significant differences were observed among the BEO, P‐BEO and Flu groups for any EPM parameter (*p* > 0.05) (Figure 4D–H).

### 3.4 Effects of BEO on Thymic and Splenic Histoarchitecture in PPD Model Rats

Given BEO's established efficacy in PPD models, we assessed its impact on thymus and spleen using H&E staining to evaluate potential systemic toxicity. Thymic morphology analysis revealed: Preserved corticomedullary demarcation; Densely populated thymocytes in cortex; Uniform basophilia without architectural distortion. Splenic architecture showed: Sharp red/white pulp boundaries; Predominant red pulp distribution; Intact lymphoid follicles in white pulp. Notably, no significant histopathological differences were observed between BEO‐treated groups (BEO and P‐BEO) and the Control or Sham groups (*p* > 0.05). These findings demonstrate that BEO inhalation at the tested dose (2.5% v/v, 1 h/day) did not induce detectable systemic toxicity in thymic or splenic tissues, supporting the safety profile of BEO administration in this PPD model (Figure 5).

**FIGURE 5:**
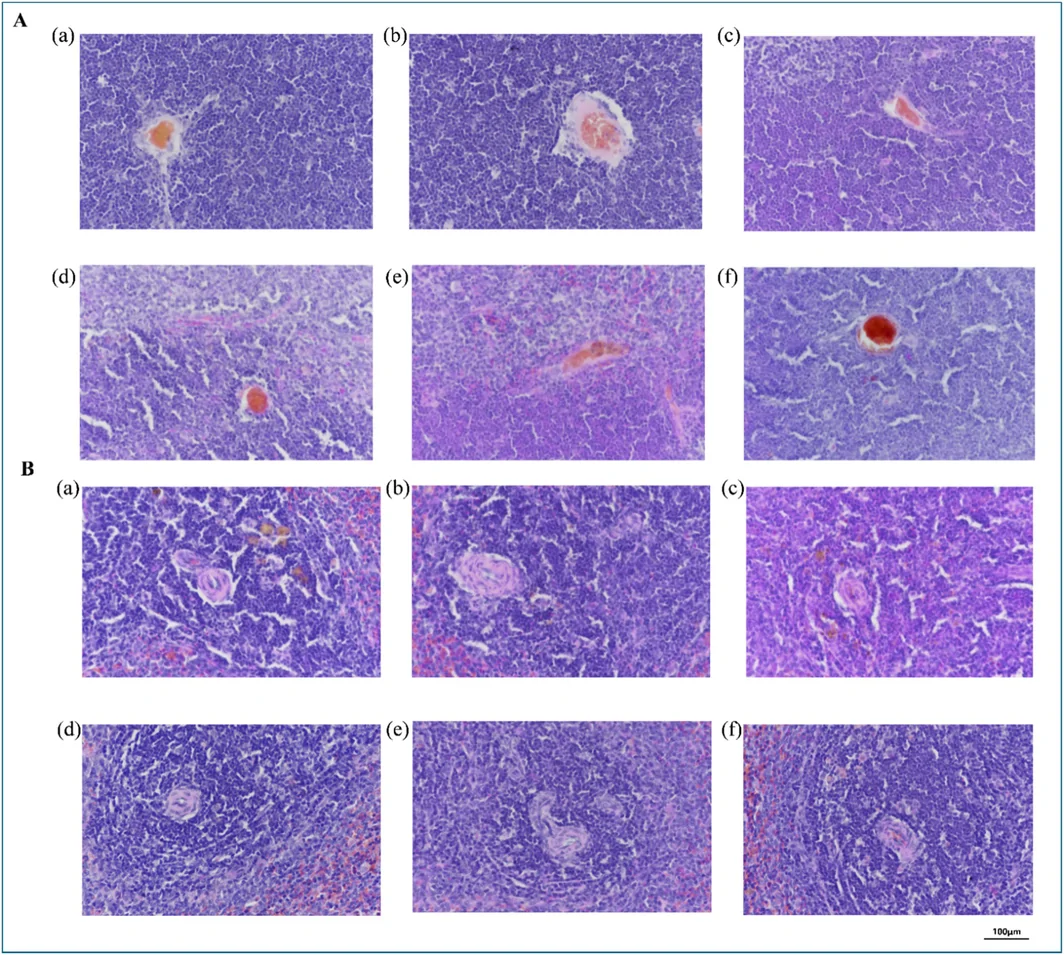
Effects of BEO on the immune organs of spleen and thymus in PPD model rats. (A) The effect of BEO on the thymus of PPD model rats (a: Control group; b: Sham group; c: PPD group; d: BEO group; e: Flu group; f: P‐BEO group). (B) The effect of BEO on the spleen of PPD model rats (a: Control group; b: Sham group; c: PPD group; d: BEO group; e: Flu group; f: P‐BEO group).

### 3.5 Molecular Docking Analysis

Molecular docking was performed to explore interactions between BEO's core components (limonene, γ‐terpinene, α‐pinene, β‐pinene, α‐phellandrene) and five target proteins (PSD‐95, SYP, IL‐1β, IGF‐1, IL‐10). The repeated ID numbers in the results are due to the fact that small molecules generate multiple conformations during pre‐processing and dock with corresponding proteins separately (Figure 6). The results indicated low binding energy for the core components against the target proteins, suggesting strong affinity and spontaneous binding (Figure 6).

**FIGURE 6:**
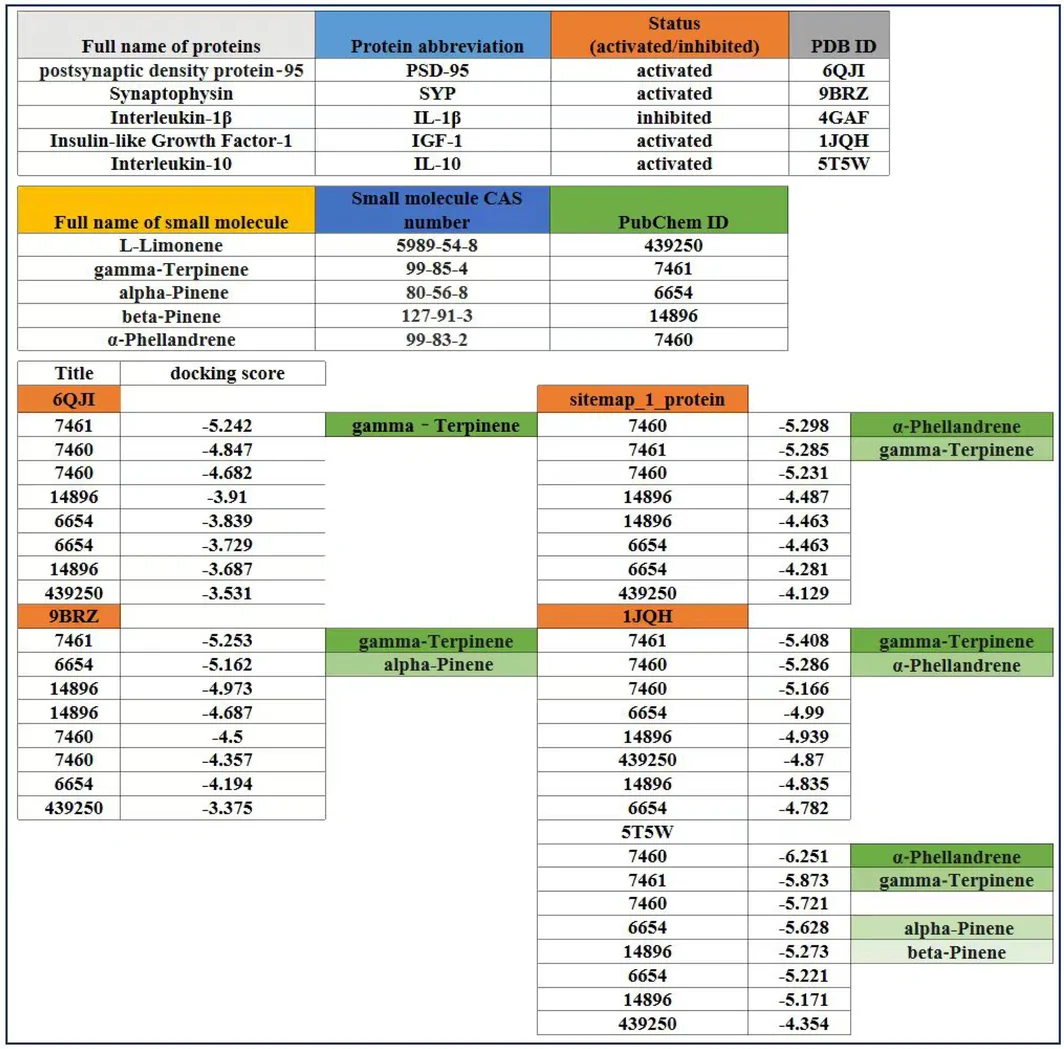
To investigate the interactions between the core active components of BEO (limonene, γ‐terpinene, α‐pinene, β‐pinene, α‐phellandrene) and five key disease‐related proteins (PSD‐95, SYP, IL‐1β, IGF‐1, IL‐10), we conducted a molecular docking analysis.

### 3.6 Inhaled BEO Enhances Hippocampal Neuronal Plasticity in PPD Rats

#### 3.6.1 BEO Restores Nissl Substance Density in PPD Hippocampus

Depression severity inversely correlates with Nissl substance abundance. Nissl staining revealed a significant reduction in Nissl body counts in the CA1, CA2, CA3 and DG regions of the hippocampus in PPD rats compared to the Control group (*p* < 0.05). Conversely, the P‐BEO, BEO and Flu groups showed a significant increase in Nissl body numbers across all hippocampal regions relative to the PPD group (*p* < 0.05). No significant difference was observed between the BEO group and the Flu group in any hippocampal subregion (*p* > 0.05). Additionally, the number of Nissl bodies in the P‐BEO group did not differ significantly from that in the BEO group (*p* > 0.05 for all regions), indicating that prophylactic BEO administration achieved comparable neuroplasticity restoration to therapeutic BEO administration (Figure 7).

**FIGURE 7:**
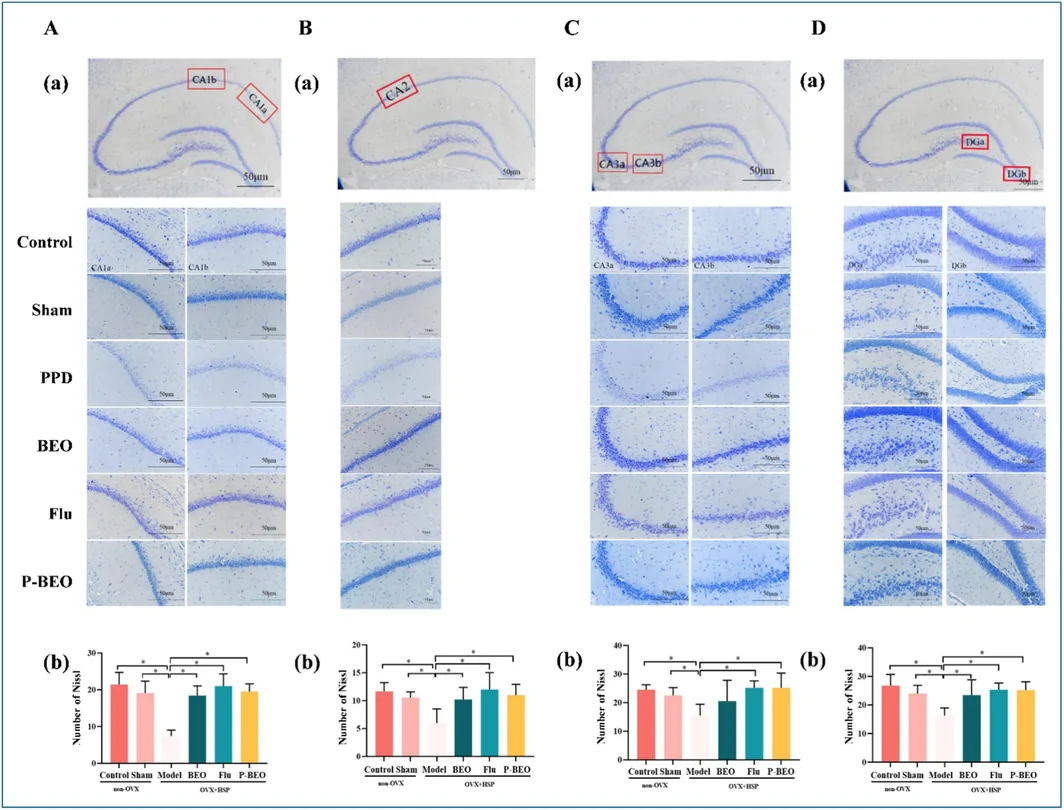
Effect of BEO on the number of hippocampal Nissl bodies in PPD model rats. (A) The effect of BEO on the number of Nissl bodies in the hippocampal CA1 region of PPD model rats. (B) The effect of BEO on the number of Nissl bodies in the hippocampal CA2 region of PPD model rats. (C) The effect of BEO on the number of Nissl bodies in the hippocampal CA3 region of PPD model rats. (D) The effect of BEO on the number of Nissl bodies in the hippocampal DG region of PPD model rats (*p < 0.05). (a) Representative histological image; (b) corresponding statistical analysis.

#### 3.6.2 Effects of BEO on PSD‐95 and SYP Protein Abundance

IHC was performed to evaluate PSD‐95 and SYP protein abundance in hippocampal subregions (CA1, CA2, CA3 and DG).

*PSD‐95*: Versus the Control group, the PPD group exhibited significantly reduced PSD‐95 protein abundance in all hippocampal subregions examined (CA1, CA2, CA3 and DG; *p* < 0.05). The BEO group showed significantly upregulated PSD‐95 protein abundance in CA1 and DG (*p* < 0.05), with an upward (non‐significant) trend in CA2 and CA3 (*p* > 0.05). The P‐BEO group displayed significantly increased PSD‐95 protein abundance across all regions (CA1–CA3 and DG; *p* < 0.05). No significant differences in PSD‐95 protein abundance were observed between the BEO and Flu groups in any region (*p* > 0.05) (Figure 8).

**FIGURE 8:**
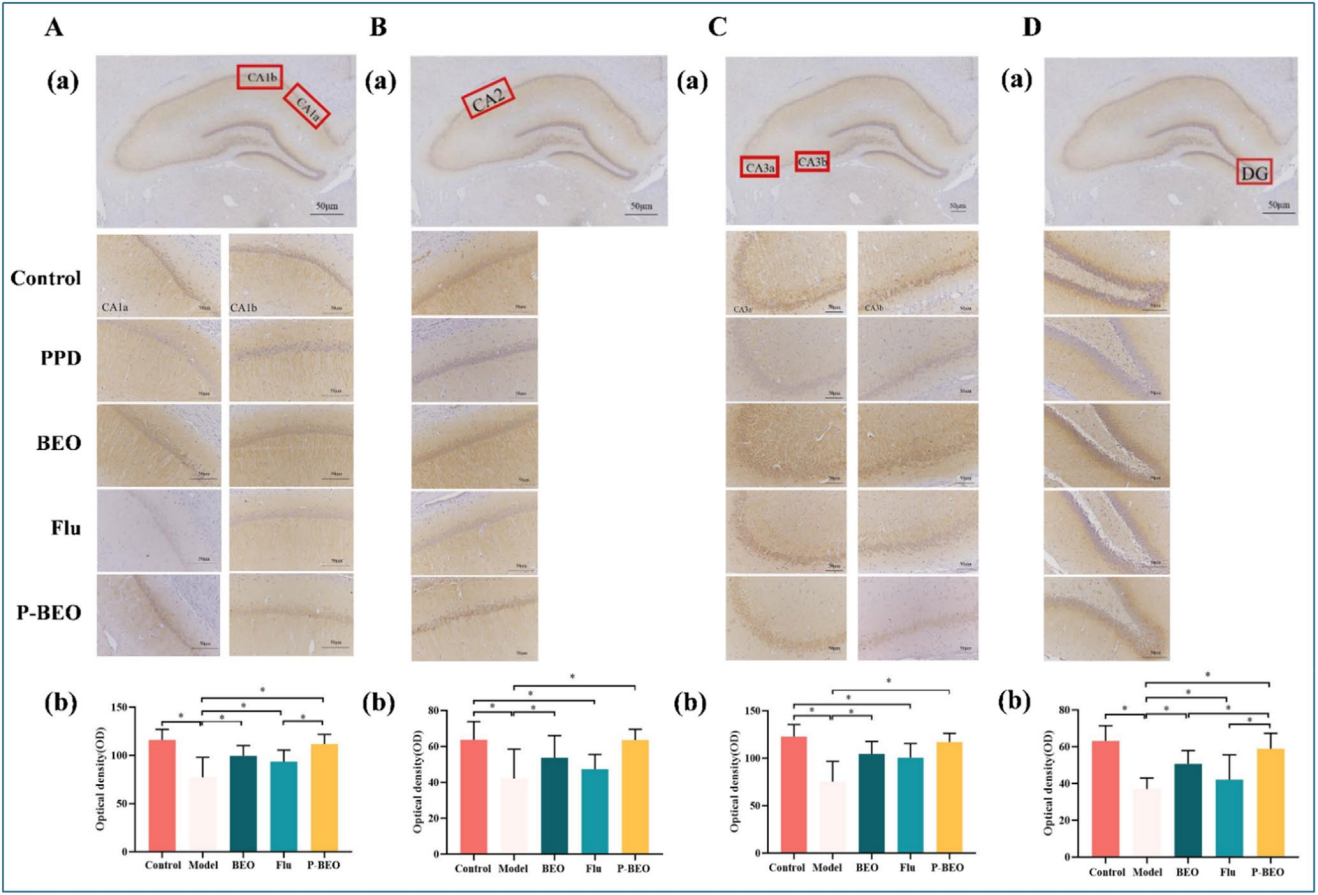
The effect of BEO on the key protein PSD‐95 in hippocampal neural plasticity of PPD model rats. (A) The effect of BEO on the expression of PSD‐95 in the hippocampal CA1 region of PPD model rats. (B) The effect of BEO on the expression of PSD‐95 in the hippocampal CA2 region of PPD model rats. (C) The effect of BEO on PSD‐95 expression in the hippocampal CA3 region of PPD model rats. (D) The effect of BEO on the expression of PSD‐95 in the hippocampal DG region of PPD model rats (*p < 0.05). (a) Representative histological image; (b) corresponding statistical analysis.

*SYP*: SYP protein abundance was significantly reduced in all hippocampal regions of the PPD group versus Control (*p* < 0.05). The BEO, Flu and P‐BEO groups all exhibited significantly increased SYP expression versus PPD group (*p* < 0.05). Notably, the BEO and P‐BEO groups showed higher SYP expression in CA1 and CA2 compared to Flu group (*p* < 0.05), while no significant differences in CA3 or DG (*p* > 0.05) (Figure 9).

**FIGURE 9:**
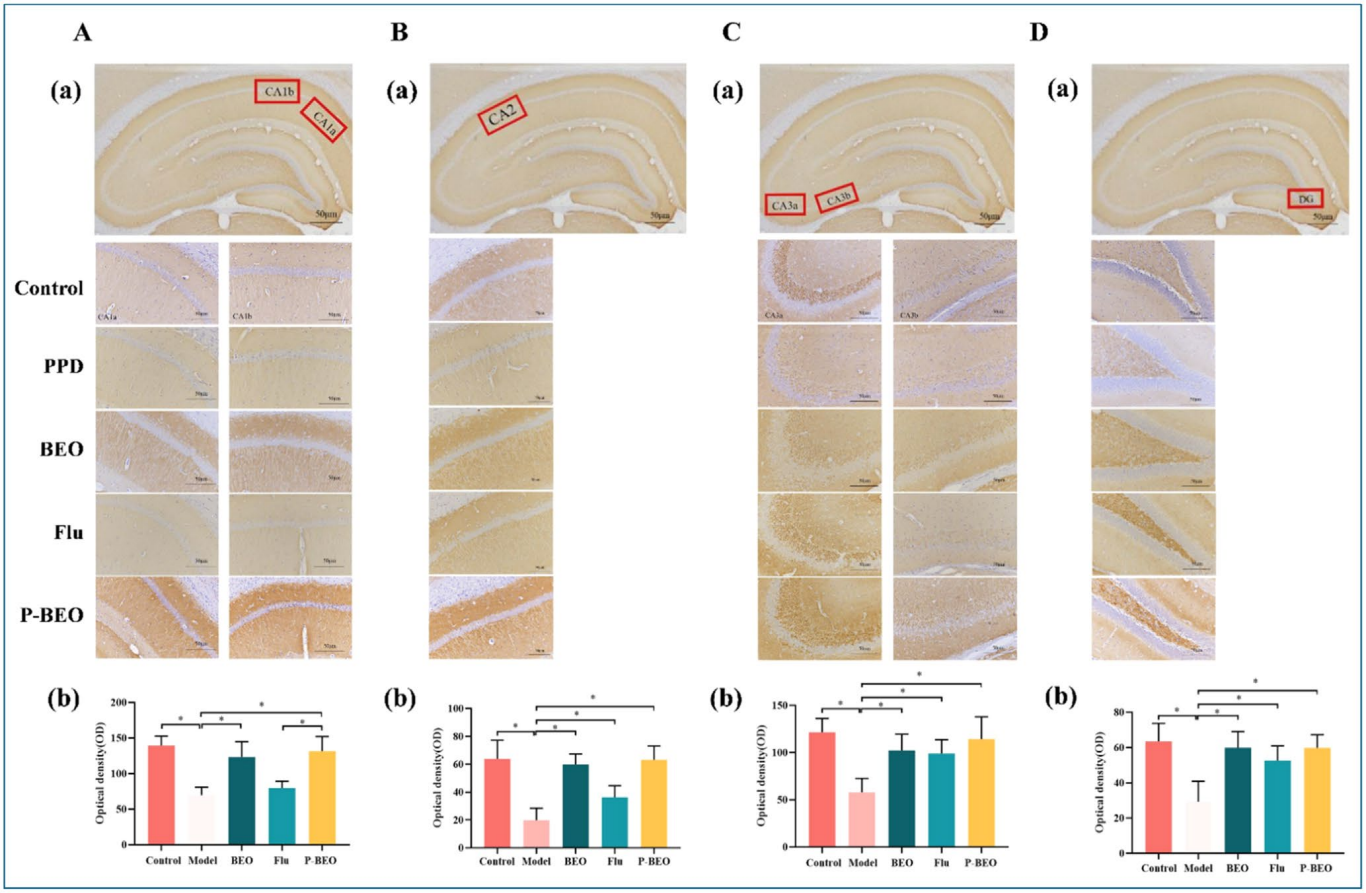
The effect of BEO on the key protein SYP of hippocampal neural plasticity in PPD model rats. (A) The effect of BEO on the expression of SYP in the hippocampal CA1 region of PPD model rats. (B) The effect of BEO on SYP expression in the hippocampal CA2 region of PPD model rats. (C) The effect of BEO on the expression of SYP in the hippocampal CA3 region of PPD model rats. (D) The effect of BEO on the expression of SYP in the hippocampal DG region of PPD model rats (*p < 0.05). (a) Representative histological image; (b) corresponding statistical analysis.

Western blot analysis further confirmed these findings. PPD group had significantly decreased PSD‐95 and SYP levels versus Control (*p* < 0.05). Flu and P‐BEO groups exhibited significantly elevated PSD‐95 and SYP (*p* < 0.05). BEO group showed increased PSD‐95 (*p* < 0.05) but only a non‐significant rise in SYP (*p* > 0.05). No significant differences in PSD‐95/SYP were observed between BEO and Flu groups (*p* > 0.05) (Figure 10).

**FIGURE 10:**
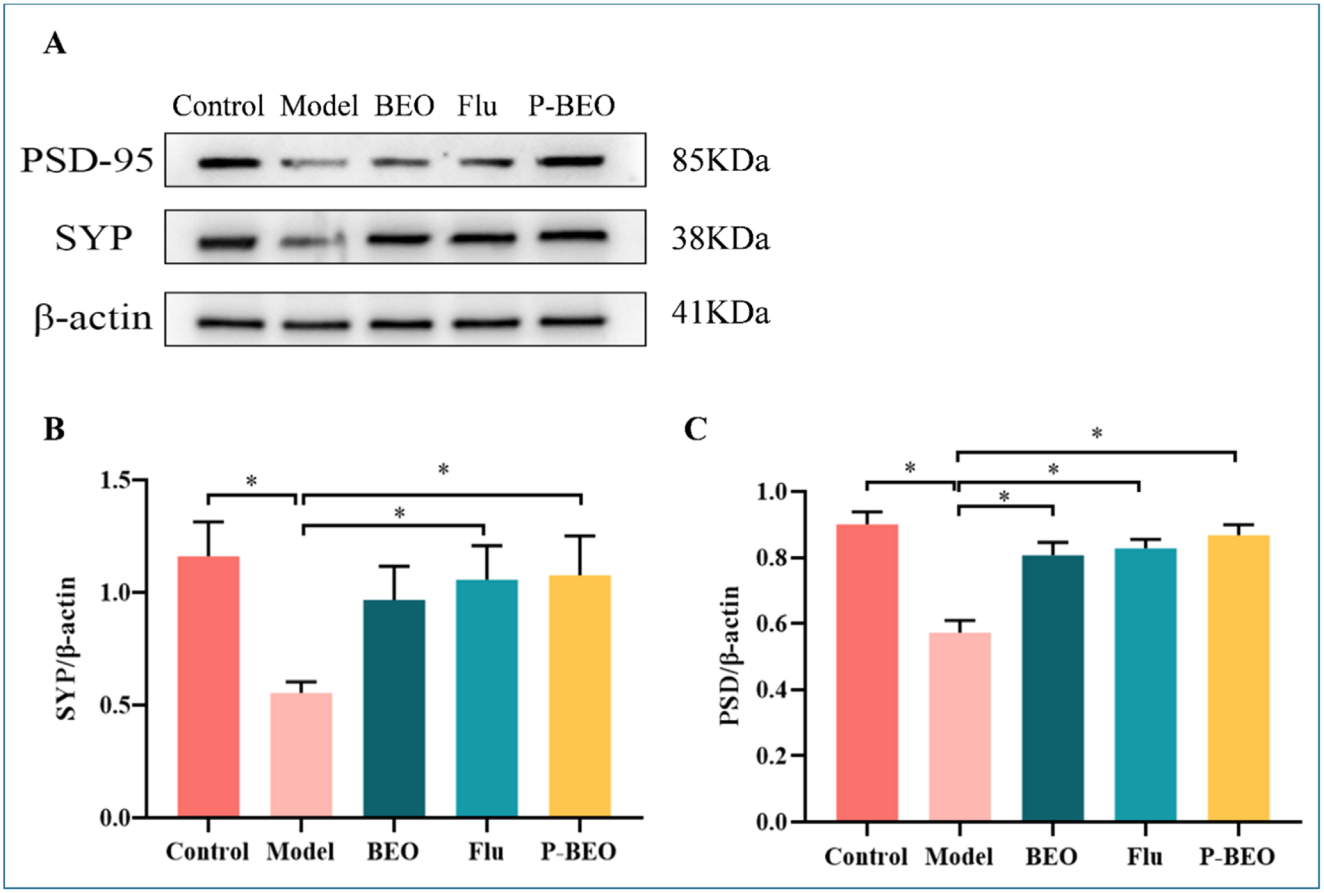
WB method was used to detect the levels of key proteins PSD‐95 and SYP in the hippocampus of rats in each group. (A) Western blot results. (B) The effect of BEO on the expression level of SYP in the hippocampus of PPD model rats. (C) The effect of BEO on the expression level of PSD‐95 in the hippocampus of PPD model rats (*p < 0.05).

### 3.7 Effects of BEO on Biomarker Levels (IGF‐1, IL‐1β, IL‐10)

Building on evidence that BEO enhances hippocampal neuroplasticity in PPD model rats, this study used ELISA to quantify IGF‐1, IL‐1β and IL‐10 levels in serum, CSF and HIPP. The goal was to determine whether BEO's antidepressant effects involve regulating these biomarkers to influence neuroplasticity.

*IGF‐1 Levels*: The PPD group exhibited significantly reduced levels of IGF‐1 in both serum and CSF compared to the Control group (*p* < 0.05). In contrast, the BEO and P‐BEO groups demonstrated significantly elevated serum and CSF IGF‐1 levels compared to the PPD group (*p* < 0.05). Notably, the P‐BEO group surpassed the levels observed in the Flu group (*p* < 0.05). No significant changes in IGF‐1 levels were observed in the HIPP (*p* > 0.05) (Figure 11A–C).

**FIGURE 11:**
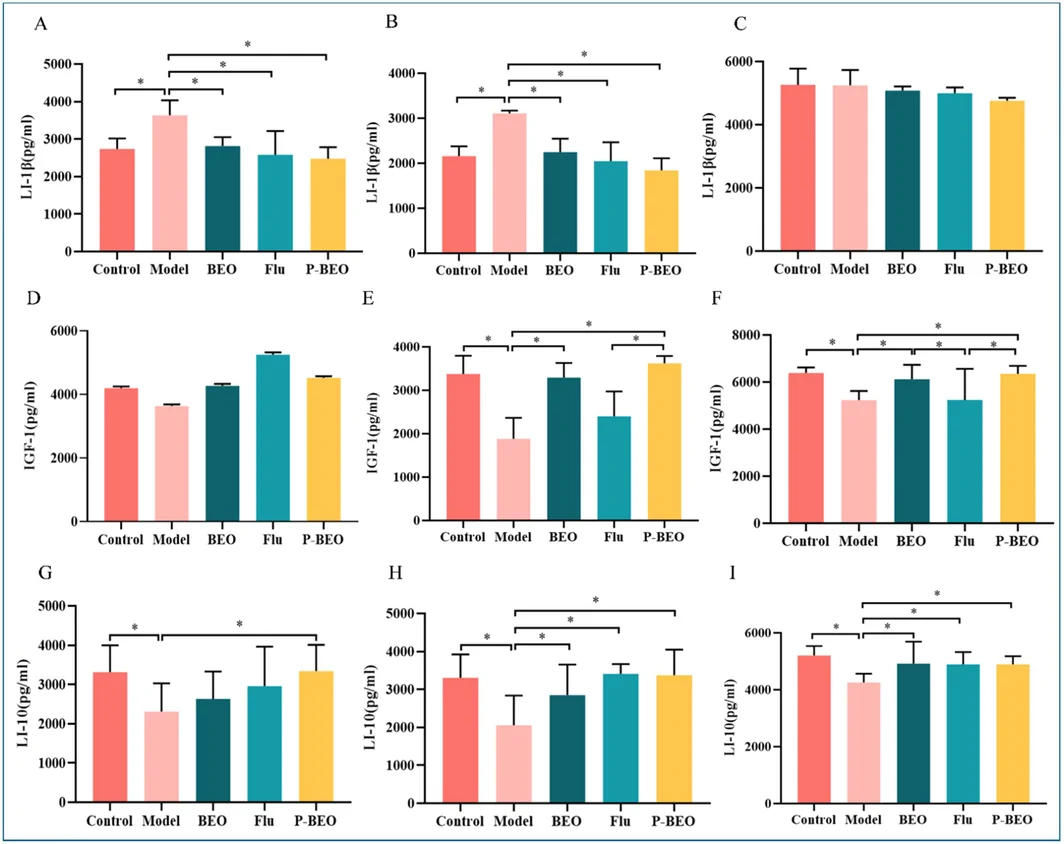
Effects of BEO on IGF‐1, IL‐1 β and IL‐10 in PPD rats. (A) The effect of BEO on the expression of IL‐1 β in the hippocampus of PPD model rats. (B) The effect of BEO on the expression of serum IL‐1 β in PPD model rats. (C) The effect of BEO on the expression of IL‐1 β in cerebrospinal fluid of PPD model rats. (D) The effect of BEO on the expression of IGF‐1 in the hippocampus of PPD model rats. (E) The effect of BEO on serum IGF‐1 expression in PPD model rats. (F) The effect of BEO on the expression of IGF‐1 in cerebrospinal fluid of PPD model rats. (G) The effect of BEO on IL‐10 expression in the hippocampus of PPD model rats. (H) The effect of BEO on serum IL‐10 expression in PPD model rats. (I) The effect of BEO on IL‐10 expression in cerebrospinal fluid of PPD model rats (*p < 0.05).

*IL‐1β Levels*: The PPD group showed a significant increase in IL‐1β levels in both the hippocampus and serum compared to the Control group (*p* < 0.05). The BEO, P‐BEO and Flu groups exhibited a reduction in IL‐1β levels in these compartments relative to the PPD group (*p* < 0.05) (Figure 11D–F).

*IL‐10 Levels*: A significant decrease in CSF IL‐10 levels was observed in the PPD group compared to the Control group (*p* < 0.05). BEO, Flu and P‐BEO interventions all resulted in elevated CSF IL‐10 levels compared to the PPD group (*p* < 0.05) (Figure 11G–I).

## 4 Discussion

PPD represents the most prevalent perinatal complication, exerting detrimental effects on both maternal and infant outcomes [46]. Globally, PPD prevalence reaches 17.2%, with a significant increase observed in China (rising from 14.8% to 20.2% between 2010 and 2020) [47, 48]. In high‐income nations such as the United States, United Kingdom and Ireland, PPD‐associated suicide has emerged as the principal cause of direct maternal death within the first year following childbirth [49]. Furthermore, PPD elevates infants' risk of developmental delays during their first year, specifically affecting social interaction, language acquisition and fine motor skills [50]. Given this substantial disease burden, developing timely and effective interventions has become imperative in perinatal medicine [51].

Currently, selective serotonin reuptake inhibitors (SSRIs) constitute the first‐line pharmacotherapy for moderate‐to‐severe depression [52]. The standard management for PPD largely relies on antidepressants approved for major depressive disorder outside the perinatal context, among which SSRIs are most commonly prescribed [53]. However, SSRIs lack rapid onset of action and are not PPD‐specific; moreover, many PPD patients exhibit suboptimal responses to SSRI therapy [54]. Kondo et al. demonstrated that 5‐hydroxytryptamine type 3 receptor (5‐HT3R) specifically regulates hippocampal IGF‐1 expression, thereby promoting neurogenesis [55]—a mechanism entirely independent of traditional SSRI pathways. These findings provide novel insights for developing rapid‐acting, PPD‐specific therapeutics. Our prior animal studies confirmed that BEO inhalation significantly upregulates: (a) serum 5‐HT levels, (b) serum IGF‐1 concentrations and (c) CSF IGF‐1 levels, while ameliorating depressive behaviours in rats subjected to CUMS [21]. Clinically, geriatric depression patients receiving daily 30‐min BEO inhalation for 16 days showed increased plasma 5‐HT and notably lower Hamilton Depression Rating Scale (HAMD) scores [56]. These collective findings establish a theoretical and empirical foundation for developing safe, rapidly‐acting BEO interventions against PPD.

The primary industrial application of bergamot ( *Citrus bergamia* Risso) fruit is for essential oil production, derived via cold‐pressing of the fruit peel [57]. BEO possesses a well‐documented history of industrial and medicinal use, being monographed in major pharmacopoeias including the European Pharmacopoeia (EP 11.0) and American Herbal Pharmacopoeia (AHP) [58]. For decades, BEO aromatherapy has been utilized in Western medicine for disease management [59] and represents an effective adjunctive intervention for mental health improvement [19]. Aromatherapy‐induced adverse effects are minimal and substantially milder than those associated with psychotropic drugs for mood disorders [60, 61]. Notably, lavender oil and BEO are prominent aromatherapeutic agents for alleviating depressive symptoms [60, 61]. Clinical evidence demonstrates that inhaled sprays containing BEO and lavender oil significantly reduce depression, anxiety and stress in geriatric populations [18]. The neurobiological mechanisms underlying inhaled aromatherapy primarily involve dual pathways: olfactory stimulation via the nasal epithelium and respiratory absorption into systemic circulation [62]. This integrated signalling elicits profound neurophysiological calming effects, effectively mitigating symptoms of anxiety and depression. A key advantage of inhalation delivery is the direct nose‐to‐brain transport via the olfactory route, bypassing the blood–brain barrier (BBB) and enabling targeted modulation of limbic structures (amygdala, hippocampus), thalamus and prefrontal cortex—critical regions for emotional regulation [63]. BEO constituents are efficiently absorbed through olfactory pathways following inhalation, achieving rapid brain distribution [64]. Modern pharmacological evidence confirms BEO's capacity to cross the BBB, demonstrating exceptional permeability, rapid metabolic clearance and favourable safety profile, which properties collectively underpin its clinical efficacy against mild‐to‐moderate depression [65]. Critically, in our PPD model rats, nebulized BEO administration markedly reduced depressive‐like behaviours.

Prenatal mood disorders (encompassing depression and anxiety) are established predictors of PPD. A recent meta‐analysis confirmed a significant association between prenatal mood disturbances and PPD onset among Chinese women [22]. This correlation was further validated in a retrospective cohort study of 1418 Chinese parturients, which identified prenatal mood disorders as one of five independent risk factors for PPD [23]. Current evidence supports the clinical utility of prenatal psychological screening and early intervention for PPD prevention. Prophylactic PPD interventions demonstrate cost‐effectiveness advantages and substantial potential for improving maternal mental health outcomes [66]. Notably, two systematic reviews with meta‐analyses established that cognitive behavioural therapy (CBT) during pregnancy significantly reduces postpartum depressive symptoms [67, 68]. Our experimental findings reveal that in PPD model rats: Preventive BEO nebulization (administered during HSP modelling) and therapeutic BEO administration (initiated after modelling), both significantly attenuated depressive‐like behaviours (*p* < 0.05 vs. model group). Crucially, histopathological analysis showed no significant differences in thymic and splenic indices between BEO‐treated groups and untreated controls (*p* > 0.05), indicating minimal systemic toxicity. These results not only validate BEO's prophylactic efficacy but also support its translational potential as a perinatal intervention given its favourable safety profile.

The hippocampus, a brain region marked by high plasticity and vulnerability to stress, plays critical roles in memory consolidation and emotional regulation [69]. Adult hippocampal neurogenesis involves generating new neurons that integrate into functional circuits via synaptic connections [69]. Synapses serve as essential conduits for interneuronal communication. Crucially, suppressed synaptic plasticity induces depressive symptomatology and cognitive impairment [70], while its disruption promotes neuronal degeneration in the hippocampus and cortex [71]. Reduced Nissl bodies in hippocampal neurons and hippocampal inflammatory responses have been observed in a rat model of hypertension‐related depression [72]. A reduction in Nissl bodies was observed in mice with post‐stroke depression [73]. Nissl bodies are primary structures involved in protein synthesis, balancing neurotransmitter levels and maintaining neuronal excitability stability [74]. Consequently, damage to Nissl substance signifies neuronal injury and is strongly correlated with compromised neuroplasticity [75].

Hormonal dysregulation induces chronic inflammation that impairs synaptic plasticity, contributing to PPD pathogenesis [11, 76]. Mechanistically, suppressing neuroinflammation and enhancing synaptic plasticity effectively prevent PPD [77]. As a fundamental neurobiological process, synaptic plasticity underpins the perception, integration and storage of complex information [73]. Crucially, it mediates adaptive responses to metabolic stress, inflammatory cues and apoptotic signals [73]. Key molecular mediators include PSD‐95, synaptophysin‐1 (SYN1) and SYP, which regulate synaptic plasticity at transcriptional and translational levels [78]. Mounting evidence indicates neuroinflammation directly modulates synaptic plasticity [79, 80]. PSD‐95, which is a scaffold protein in the postsynaptic density, helps regulate synaptic transmission, the formation of plasticity and the development of excitatory synapses [24, 25]. SYP significantly contributes to synaptic plasticity, the operation of synaptic vesicles and the release of neurotransmitters [26]. Hippocampal injury induces synaptic plasticity impairments characterized by reduced PSD‐95, SYN1 and SYP expression [27]. Inhalation of BEO increased Nissl body counts in the HIPP of CUMS‐induced rats, while HIPP plasticity‐related proteins (PSD‐95, SYP) were significantly upregulated [15]. Our results indicate that inhalation of BEO in the P‐BEO group and BEO group significantly increased the number of Nissl bodies, as well as the expression of PSD‐95 and SYP proteins, in the hippocampus of PPD rats.

In 2019, the FDA approved brexanolone as the first medication specifically indicated for PPD [81]. The need for a 60‐h intravenous infusion was highlighted in clinical trials, which demonstrated quick and sustained symptom relief, with therapeutic benefits lasting at least 9 months after delivery [13, 82, 83]. Common adverse reactions include flushing (28%), xerostomia (21%), somnolence (15%) and transient syncope (3%; duration < 90 min) [53]. According to the FDA Risk Assessment and Mitigation Strategy, medication must be administered in medical facilities equipped with emergency equipment, and continuous pulse oximetry and sedation scale monitoring must be provided every 2 h [53]. In addition, brexanolone treatment is expensive, with an average cost of about $36,000, mainly obtained by married white women with private insurance [13]. These challenges make it difficult for many in need to obtain brexanolone. In 2023, FDA approved zuranolone as the first oral PPD agent [49, 84]. However, methodological limitations in pivotal trials (e.g., open‐label design, high placebo response) have raised efficacy concerns [85]. The latest meta‐analysis confirms its effectiveness in improving depression and anxiety, but the incidence of adverse events is higher than with placebo, including somnolence, dizziness, sedation and headache [86]. Additionally, according to the FDA's Clinical Trial Safety Data Analysis Report, in a Phase III clinical trial targeting major depressive disorder (MDD), the incidence of suicidal ideation or suicidal behaviour in the zuranolone group (1.0%) was higher than in the placebo group (0%) [87], a phenomenon not observed in PPD patients [88]. These neuroactive steroids (NAS) represent a novel therapeutic class for PPD [89]. Mechanistic investigations reveal that brexanolone significantly suppresses pro‐inflammatory cytokines—TNF‐α, IL‐6 and IL‐1β [14, 15, 16], suggesting that the antidepressant efficacy of neuroactive steroids (NAS) may be mediated through anti‐inflammatory pathways [90]. These discoveries not only provide novel pathophysiological insights into PPD pathogenesis but also illuminate strategic directions for developing safer, orally bioavailable and cost‐effective therapeutic regimens.

Accumulating evidence indicates immune dysregulation in PPD patients, with neuroinflammation emerging as a pivotal pathophysiological mechanism [33, 34, 35]. Inflammatory and immune perturbations play central roles in depression pathogenesis, positioning anti‐inflammatory strategies as promising therapeutic avenues [91, 92]. Empirical data demonstrate that elevated pro‐inflammatory cytokines (e.g., IL‐6, IL‐1β, TNF‐α) are elevated in PPD women—indicating that postpartum hyperinflammation constitutes a critical pathophysiological driver of depressive symptomatology [93]. Elevated levels of pro‐inflammatory cytokines in the peripheral blood of patients with PPD may compromise the integrity of the BBB by activating astrocytes and diminishing transendothelial resistance. This disruption facilitates the infiltration of peripheral immune cells or cytokines into the brain, potentially inducing neuroinflammation and subsequently contributing to the onset of depressive symptoms.

Elevated IGF‐1 levels are documented in cases of severe depression [94, 95, 96]. Murine models demonstrate antidepressant‐like effects following subcutaneous or intracerebroventricular IGF‐1 administration [30, 31, 32]. Despite IGF‐1's ability to cross the BBB [97], rapid clearance from CSF limits its central bioavailability [98]. The Japan Environment and Children's Study revealed that pregnant women with high serum IGF‐1 concentrations in early pregnancy had a lower likelihood of developing PPD compared to those with low levels [28]. This protective association aligns with the Pomeranian Health Study [29]. These observations suggest that elevated serum IGF‐1 concentrations in early pregnancy may play a role in preventing the onset of PPD. Critically, our study found that BEO in the P‐BEO group and BEO group significantly upregulates IGF‐1 levels in serum and CSF; compared with the fluoxetine group, IGF‐1 levels in serum and CSF were significantly increased in the P‐BEO group. Mechanistically, BEO's dual‐phase benefits mirror the anti‐inflammatory action of neurosteroids (e.g., brexanolone's suppression of TNF‐α/IL‐6/IL‐1β [14, 15, 16]), yet achieve this via natural constituents with minimal adverse effects. The IGF‐1 upregulation in P‐BEO rats (*p* < 0.05 vs. Flu) is particularly salient, as low early‐pregnancy IGF‐1 predicts PPD in humans [28, 29]. This supports BEO's unique potential as both a preventive agent (modifying biomarker trajectories) and acute intervention.

Previous research indicates that IL‐1β levels show a significant positive correlation with the severity of perinatal depression, implicating early postpartum IL‐1β surge as a key risk factor for PPD pathogenesis [16, 36]. Recent single‐cell RNA sequencing analyses revealed increased cDC2 dendritic cells and proliferative B cells in PPD patients' peripheral blood—positively correlating with IL‐1β expression [99]. Animal studies further suggest IL‐1β modulation represents a therapeutic target [99]. Mechanistic investigations establish IL‐1β as a pivotal inflammatory mediator exhibiting hippocampus‐specific overexpression, playing a key role in PPD pathogenesis by regulating neuroinflammatory responses and synaptic plasticity [100]. Excessive IL‐1β can disrupt the BBB [101, 102, 103] and increase its paracellular permeability [104, 105]. This increased permeability facilitates the entry of peripheral pro‐inflammatory cytokines and immune cells to enter the brain parenchyma, causing neuroinflammation, neuronal dysfunction and ultimately depression [106, 107, 108]. IL‐1β is posited to be a key pro‐inflammatory cytokine that exerts direct neurotoxic effects on the adult HIPP [109]. In animal models, the administration of IL‐1β or its inducers (such as LPS and IFN‐α) has been reported to inhibit HIPP function and exacerbate depressive‐like behaviours [110, 111]. Fu et al. found that preventing the elevation of pro‐inflammatory factors like IL‐1β in the HIPP of PPD mice could protect/enhance synaptic plasticity to prevent PPD [112]. Our study found that compared to the HSP group, IL‐1β expression in serum and HIPP was significantly reduced in the BEO, Flu and P‐BEO groups.

Inflammatory factors play a crucial role in many diseases [113, 114]. Human studies associate elevated IL‐1β and reduced IL‐10 expression in postpartum plasma/serum/whole blood with the manifestation of depressive symptoms at 4–6 weeks postpartum [115]. During pregnancy, IL‐10 levels increase versus non‐pregnant states [116] but decline post‐delivery [117]. Currently, a nested case–control study using plasma samples from 247 women with PPD and 243 age‐matched controls has provided comprehensive perinatal cytokine profiling, which confirmed a reduction in plasma IL‐10 levels during both pregnancy and the postpartum period in individuals with PPD [37]. IL‐10 appears to be a key molecule consistently positively associated with the risk of perinatal emotional disorders in humans [118]. Cytokine changes during pregnancy are not only observed in peripheral blood but also in the brains of animal models, with IL‐10 levels in the postpartum hippocampus (HPC) also increasing [119]. Additionally, non‐pregnant female mice with IL‐10 knockout have been shown to exhibit characteristics of depressive symptoms, which were improved by IL‐10 administration [120]. Thus, IL‐10 dysregulation in PPD likely involves both systemic and central compartments, implicating its pathogenic role. Critically, our data confirm BEO significantly upregulates IL‐10 in rat CSF in both the PBEO and BEO groups.

Only by understanding the pathogenic factors of diseases can we better adjust and choose treatment methods [121, 122]. Current first‐line pharmacotherapies for PPD face significant limitations. Crucially, both lack prophylactic utility for high‐risk populations. In stark contrast, our study demonstrates that non‐invasive BEO inhalation not only matches fluoxetine's therapeutic efficacy (*p* > 0.05) but also offers dual advantages: Preventive potential: Prophylactic administration (P‐BEO) during simulated pregnancy significantly outperformed fluoxetine in elevating IGF‐1 (*p* < 0.05)—a biomarker linked to reduced PPD risk in humans [28, 29]. Safety and accessibility: No systemic toxicity (validated by thymic/splenic histology) and low‐cost aromatherapy delivery align with WHO recommendations for scalable perinatal interventions [51]. To our knowledge, this is the first study to establish a dual‐phase BEO intervention strategy (prophylactic + therapeutic) for PPD. Our innovative prophylactic approach (P‐BEO group) administered during hormone‐simulated pregnancy successfully prevented synaptic deficits (↑PSD‐95/SYP/Nissl bodies) and suppressed neuroinflammation (↓IL‐1β, ↑IL‐10)—effects comparable to post‐onset treatment. This preventive efficacy, combined with BEO's direct nose‐to‐brain delivery bypassing the BBB [63, 64, 65], positions it as a practical candidate for prenatal PPD prevention in high‐risk women, particularly where pharmacotherapy access is limited.

GC–MS analysis of the BEO identified 15 constituents, with the major components being limonene (61.69%), γ‐terpinene (28.02%), α‐pinene (3.52%), β‐pinene (2.80%) and α‐phellandrene (1.28%). To explore potential interactions between BEO's core active components (limonene, γ‐terpinene, α‐pinene, β‐pinene and α‐phellandrene) and five key proteins associated with depression (PSD‐95, SYP, IL‐1β, IGF‐1 and IL‐10), molecular docking analysis was performed. Interestingly, γ‐terpinene was the only component that successfully docked with all five target proteins. Several essential oils, including Hirami lemon essential oil (CDE), oregano essential oil (OEO) and *Picea mariana* essential oil (PMEO), have been reported to exhibit antidepressant effects. According to GC–MS spectrometry, OEO is composed mainly of thymol (20.72%), gamma‐terpinene (γ‐terpinene, 8.83%), borneol (8.72%), cymene (6.83%), carvacrol (6.27%), alpha‐terpinene (5.26%) and sabinene (4.92%) [123]. Another study found that the CDE of *Citrus reticulata* var. *depressa* contains eight major compounds, with d‐limonene (45.71%), γ‐terpinene (34.65%), linalool (6.46%), *p*‐cymene (2.57%), α‐terpineol (2.04%), α‐pinene (1.89%), α‐terpinolene (1.46%) and β‐pinene (1.16%) [124]; in a similar vein, the principal active constituents of PMEO are bornyl acetate (44.95%), γ‐terpinene (14.17%) and β‐pinene (10.12%) [125], which can find through comparison that gamma terpenes are the second main component contained in these essential oils. Notably, γ‐terpinene is a monoterpene compound that may influence mood through direct actions on the central nervous system and olfactory pathways [126]. Its consistent presence across these essential oils with documented antidepressant properties suggests that γ‐terpinene may be a key constituent contributing to their efficacy. Therefore, further validation of this single component from BEO is warranted to support the development of novel antidepressants.

Our study has several limitations. The HSP with abrupt withdrawal model was used to establish PPD rats. While widely adopted, this approach incompletely recapitulates the multifactorial aetiology of human PPD. In the future, we can combine genetic susceptibility models (such as 5‐HTTLPR gene knockout) and social stress models to construct animal models that are closer to human PPD, thereby improving the external validity of the study. Since the PPD rat model requires ovariectomy, we did not conduct teratogenicity tests during the rat pregnancy period to verify the safety of preventive inhalation of BEO. Future studies could extend the observation period and combine toxicological experiments to comprehensively evaluate its safety. Although GC–MS characterized BEO's principal bioactive constituents, the mechanistic actions of individual components remain unexplored. Future studies focusing on the identification of key active compounds and their molecular targets would further clarify the mechanisms underlying BEO's antidepressant effects and support its clinical translation.

## 5 Conclusion

This study preliminarily confirms that BEO alleviates PPD by regulating hippocampal synaptic plasticity and neuroinflammation. The potential mechanism may be related to increased IGF‐1 concentrations in serum and CSF, decreased IL‐1β in serum and HIPP and increased IL‐10 concentrations in CSF. This study pioneers a dual‐phase strategy for PPD management—combining prophylactic and therapeutic BEO inhalation. This study is the first to reveal that BEO, when administered prophylactically during simulated pregnancy, effectively mitigates hippocampal synaptic impairment and neuroinflammation, outperforming fluoxetine in enhancing neuroprotective IGF‐1; BEO's non‐invasive delivery circumvents limitations of current PPD drugs (e.g., brexanolone's infusion burden, zuranolone's neuropsychiatric risks). These findings validate BEO as a safe, cost‐effective candidate for clinical translation—particularly as a prenatal preventive regimen for women at high PPD risk. Future studies should prioritize human trials validating this dual‐phase approach.

## Author Contributions

**Huan Xu:** conceptualization, methodology, writing – original draft. **Shike Zhang:** conceptualization, methodology, writing – original draft. **Hui He:** conceptualization, methodology, writing – original draft. **Xue Zhao:** conceptualization, writing – original draft. **Yupei Cheng:** methodology, writing – original draft. **Yingdong Wang:** writing – review and editing, formal analysis. **Jinzhou Li:** formal analysis, writing – review and editing. **Yi Yu:** writing – review and editing, formal analysis. **Yuhong Bian:** conceptualization, methodology, writing – review and editing. **Yi Guo:** conceptualization, methodology, writing – review and editing, funding acquisition. **Mingxing Zhang:** conceptualization, methodology, writing – review and editing, funding acquisition, project administration.

## Funding

This study was supported by National key R&D Program of China (2024YFC3507302), the Key Project of National Natural Science Foundation of China (grant no. 82030125), Manned Space Engineering Space Medical Experiment Project of China (grant no. HYZHXMX02002), Henan Provincial Natural Science Foundation Youth Science Fund (Category C) Project (grant no. 262300420525) and Henan Provincial Science and Technology Research Project (grant no. 262102310498).

## Disclosure

*Provenance and Peer Review*: Not commissioned, externally peer‐reviewed.

*Generative AI Statement*: The author(s) declare that no Generative AI was used in the creation of this manuscript.

## Conflicts of Interest

The authors declare no conflicts of interest.

## Data Availability

The data that support the findings of this study are available from the corresponding author upon reasonable request.
